# Metabolomics of Rhabdomyosarcoma Cell During Echovirus 30 Infection

**DOI:** 10.1186/s12985-017-0812-7

**Published:** 2017-07-27

**Authors:** Sarika Tiwari, Tapan N. Dhole

**Affiliations:** 1 Department of Microbiology (Virology Section), Sanjay Gandhi Post Graduate Institute of Medical Sciences (SGPGIMS), Lucknow, Uttar Pradesh -226014 India; 2 Centre for Animal Disease Research and Diagnosis, Indian Veterinary Research Institute, Bareilly, UP India

**Keywords:** Echovirus 30, NMR, Cellular metabolomics, Aseptic meningitis, Tissue culture

## Abstract

**Background:**

Echovirus 30 (E30) causes acute aseptic meningitis. Viral replication requires energy and macromolecular precursors derived from the metabolic network of the host cell. The effect of viral infection within a host cell metabolic activity remains unclear.

**Methods:**

To gain an insight into cell-virus interaction during E30 infection we used a human rhabdomyosarcoma cell line. In a new approach to metabolomics, ^1H^ NMR was used to measure the level of various cellular metabolites at different times of infection and morphological examination of the cells. Statistical analysis was done by using Confidence interval (CI) 95% and One-way ANOVA test.

**Results:**

The^1^H NMR metabolite spectrum signals were observed between mock infected and virus infected cells. Both mock infected and virus infected cells utilized glucose through metabolic pathways and released metabolic end products. Upon infection, the concentration of Alanine, Lactate, Acetate, Glutamate, Tyrosine, Histidine, Phenylalanine, Creatine, Choline and Formate, increased. Interestingly, all of these augmented metabolites were decreased during later stage of infection. The cells showed wide-ranging lipid signals at the end of infection, which correlates with the morphological changes as apoptosis (programmed cell death) of cells was observed. A significant association was found between time interval (12 h, 24 h, and 48 h) and metabolites likewise Alanin, Lactate, Acetate, Glutamate, Tyrosine, Histidine, Phenylalanine, Creatine, Choline and Formate respectively released by cell during infection, which is highly significant (*p* < 0.01).

**Conclusion:**

Progressive breakdown and utilization of all cellular components were observed as the infection increased. This study is useful for monitoring the cellular metabolic changes during viral infection.

## Background

Enteroviruses (EVs) are responsible for 30,000 to 50,000 hospitalizations for aseptic meningitis per year in the United States. Echovirus 30 (E30) (genus: *Enterovirus*; family, *Picornaviridae*) is one of the most frequently isolated EVs, causing extensive outbreaks of E30 in temperate climates in several countries [1, 2].

Metabolomics is a new approach that facilitates multivariate profiling of the integrated metabolic responses of complex systems to pathophysiological stress and provides corresponding information to genomics and proteomics [3]. The use of high-resolution nuclear magnetic resonance (NMR) spectroscopy to study metabolomics in biological fluids, tissues, and cells and to investigating the biochemical consequences of human disease is rapidly increasing [4–6]. This approach is attractive in the study of cell metabolism owing to its unique non-invasive characteristics which can generate information on multiple pathways monitored simultaneously in a single step.

During viral infection in the host cell, delivery of viral nucleic acid involves a complex series of tightly regulated events where the invading virus utilizes the biosynthetic components of the host cell to reach its goal, altering cell metabolism. Depending on the host cell and virus type, different altering mechanisms have been proposed [7]. Studies on virus-cell interactions have proven to be valuable in elucidating viral-cellular processes [8–13].

Cell culture is widely used as an in vitro model due to increased experimental control of physiological parameters than tissue or in vivo studies. The widespread use of animal cells grown in tissue culture as models for investigation of properties of normal and malignant cells intensifies the importance of a clear understanding of the dynamics of cellular metabolism [14]. The current understanding of the effect of viral infection on the metabolism of all metabolites in tissue culture is still relatively inadequate.

Thus far, there have been no reports on metabolic studies of E30 infection in human cell lines using NMR spectroscopy. In this study, we use high resolution ^1^H NMR spectroscopy to investigate the effect of E30 infection during acute, sub-acute and chronic stage infections in a rhabdomyosarcoma embryonic (RD) human cell line. NMR spectroscopy can play a strategic role in this scientific challenge as it can be used to study the interactions, both from thermodynamic and kinetic points of view, between and among biomolecules and small ligands, monitoring whole metabolic processes. In addition, NMR spectroscopy can be used to solve the structure of biomolecules and tell how the biomolecules interact. Theoretically, the combination of a metabolite and an analogue capable of antagonizing its biological action at very low concentrations could provide a sensitive system for determining slight amounts of metabolites. This study investigates the interaction between E30 and human cells using NMR spectroscopy to determine the mechanistic contribution of a viral infection.

## Methods

### Preparation of cell

RD cells were obtained from the Center for Disease Control and Prevention, Atlanta, GA, USA and were cultivated in the Eagle’s minimum essential medium (MEM) (Eagle 1959) supplemented with 10% fetal bovine serum (FBS), 100 U ml^−1^ of penicillin, and 10 mg ml^−1^ of streptomycin, essential amino acids, and L-glutamine (obtained from Sigma-Aldrich Co). The cells were cultured in 75 cm^2^ flasks (Corning Inc. USA). The cell monolayer was washed twice with 1× phosphate buffered saline solution without calcium and magnesium ions (Sigma-Aldrich), then trypsinized (0.5 g trypsin; 0.2 g EDTA.4Na/l HBSS, Sigma-Aldrich) and kept at 36.5 °C in the incubator until the cells detached from the surface. Cells were counted using a hemocytometer by taking quadruplicate samples from each of three flasks using the procedure of Jenkin and Anderson [15]. Fresh medium (30 ml) was then added into the flask. The cells were subcultured at 5 to 7 day intervals.

### Virus

E30 used in this study was isolated from environmental specimens. The virus was serotyped according to World Health Organization’s protocol [16] and confirmed by RT-PCR with specific primers [17]. Virus stock was prepared by infecting RD cells in 25 cm^2^ flasks (Corning Inc. USA). Virus infected and mock infected cells were incubated at 36.5 °C with 2% MEM. After 48 h of infection, the culture was aliquoted and kept at-80 °C for further use.

### Plaque assay

Plaque assays of E30 were performed on monolayers of RD cells in 60-mm six-well tissue culture plates. Tenfold serial dilutions of virus stock were added to 90% confluent monolayer. After 1 h incubation at 36.5 °C with 5% CO_2,_ unabsorbed virus was removed by gentle washing with PBS and an overlay of fresh media (1% agarose in MEM with 1% FBS) was added. After 4 days incubation, the cells were fixed with 10% formaldehyde and stained with 0.5% crystal violet and plaques were counted.

### Virus infection

After the cells were grown with 100% confluence, the growth medium (MEM with 10% FBS) was replaced with maintenance medium (MEM with 2% FBS) from all the flasks. Three flasks were inoculated with 200 μl of virus (1 × 10 [5] Pfu/ml), while the other three flasks were inoculated with 200 μl of sterile PBS served as mock infected for each time point (12 h, 24 h, 48 h). After 12 h, one set of flasks both mock infected and virus infected was taken out from the incubator, photographs of the cells were taken under a Nikon inverted microscope at a magnification of X 400 and the maintenance medium was taken out and stored at −80 °C until 1H NMR experiments were performed for analyzing the extracellular metabolites from the medium. The cells were harvested by trypsinization, stained with trypan blue and counted, washed twice with ice-cold PBS 1X (4 °C, pH = 7. 4) and suspended in buffered D2O (50 ml PBS 10X and 450 ml D_2_O) for 1H NMR experiments of the whole cells. Virus infection in the remaining two sets of flasks was continued for 24, 48 h period and, at the end of each period, photographs of cells were taken, the maintenance medium was taken out for 1H NMR experiments and the cells were harvested, counted and suspended in buffered D2O as described above for flasks with 12 h virus infected and mock infected cells.

### Other components

Eagle’s minimum essential medium, HEPES buffer (N-2-hydroxyethylpiperazine-N0-2-ethanesulfonic acid), sodium bicarbonate, phenol red, deuterium oxide (D_2_O),trimethylsilylpropionic acid (TSP), sodium salt (all from Sigma Aldrich, USA), penicillin, streptomycin, FBS, L-Glutamine, PBS, fungizone, trypsin, trypan blue stain, (all from GIBCO, USA) and cell culture flask 75 cm^2^ (Corning, Inc. USA) were used for the study.

### NMR experiments

One and two-dimensional NMR analyses were performed on a Bruker Biospin Avance 400 MHz NMR spectrometer using a 5 mm broadband inverse probe equipped with z-gradient. Analyses were performed according to the protocol described in a previously published article Tiwari et al. in 2012 [16]. An experiment was performed in triplicates.

### Extracellular metabolites

Collected extracellular media from virus infected cells and mock infected cell counterparts were thawed. An aliquot of 0.5 ml of each medium was drawn separately into 5 mm NMR tubes and 1H NMR spectra were recorded. After inserting a TSP control capillary, all spectra comparing the metabolite patterns excreted by virus infected and mock infected cells were recorded under identical experimental conditions.

### Whole-cell metabolites

All virus infected cells (12–48 h post infection) and mock infected cells (12–48 h post infection) were washed twice with 1× PBS and suspended in buffered D_2_O (50 μl PBS 10× and 450 μl D_2_O). Cells were then transferred to 5 mm NMR tubes and 1H NMR spectra were recorded under identical experimental conditions.

### Intracellular metabolites

Water-soluble intracellular metabolites of virus infected and mock infected whole cells were extracted into PBS prepared in D_2_O (50 μl PBS 10× and 450 μl D_2_O) by sonicating the cells under ice-cold conditions. This was followed by centrifugation at 12,000 rpm at 4 °C for 10 min. The supernatants were stored for lipid extraction. The whole cell lysate was thoroughly mixed by vortexing the sample and brought up to a total volume of 0.5 ml. The whole cell lysates containing water-soluble cellular metabolites were then placed in 5 mm NMR tubes and one-dimensional ^1^H NMR spectra were recorded. Suppressing the residual water by pre-saturation allowed for spectra to be collected under identical conditions. For 48 h virus infected and mock infected cell extracts, a 1H–1H two-dimensional double-quantum filtered correlated spectroscopy (DQF-COSY) experiment was also performed by suppressing the residual water signal by pre-saturation. After recording the spectra, all samples were stored at-80 °C for lipid extraction.

### Cell lipids

Cell lysates, stored at-80 °C after recording the proton NMR spectra, were subjected to lipid extraction following Folch’s extraction procedure [18]. Supernatant and residue were lyophilized. Briefly, the residual cell pellet was mixed with 3.18 ml of chloroform, methanol and saline water in a ratio of 2.0:1.0:0.18. The resultant mixture was sonicated for 3 min under ice cold conditions and centrifuged at 2400 rpm for 10 min to allow a clear separation of the aqueous and non-aqueous phases. Subsequently, the aqueous (upper) phase was collected and subjected to re-extraction of the remaining lipids, if any, by treatment with 2.0 ml of chloroform and methanol in a ratio of 2:1, sonicated and centrifuged for 10 min. The non-aqueous (lower) phase from both extraction steps were pooled and dried using nitrogen gas. The residue obtained was dissolved in 0.5 ml CDCl_3_:CD_3_OD mixtures in a ratio of 2:1 and ^1^H NMR spectra were recorded.

### Statistical analysis

The multiple comparisons of time intervals corresponding to various chemical components (dependent variable) and their significant value were calculated. The Confidence interval (CI) 95% was also estimated. The One-way ANOVA test was performed with all time interval groups were also calculated. A significant association was found between time interval (12 h, 24 h, and 48 h) and metabolites likewise Alanin, Lactate, Acetate, Glutamate, Tyrosine, Histidine, Phenylalanine, Creatine, Choline and Formate respectively released by cell during infection, which is highly significant (*p* < 0.01).

## Results

### Microscopic observation

**Fig. 1:**
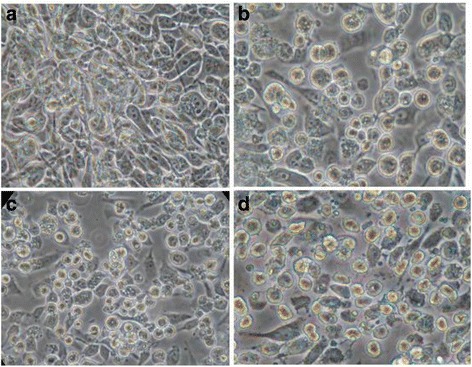
Cells in culture flasks monitored under a Nikon inverted microscope at a magnification of 400×: (**a**) mock infected RD cells; (**b**) –(**d**) RD cells infected with E30 for (**b**) 12 h, (**c**) 24 h, and (**d**) 48 h

The total cell count at 12 h post virus infection was slightly less when compared with mock infected cells under similar conditions. However, no morphological changes were seen at 12 h infection when compared with mock infected cells as observed under the microscope (Fig. 1b). At 24 h (Fig. 1c) and 48 h post infection (Fig. 1d), cytopathic effects are visible in virus infected cells when compared with mock infected control cells (Fig. 1a). At 24 h post infection, detachment of cells from the flask surface was observed (Fig. 1c). This detachment increased with time. At 48 h, all virus infected cells detached from the flask surface and showed morphological changes (rounding of cells) (Fig. 1d), whereas no morphological changes were seen in mock infected cells.

### NMR analysis

**Fig. 2:**
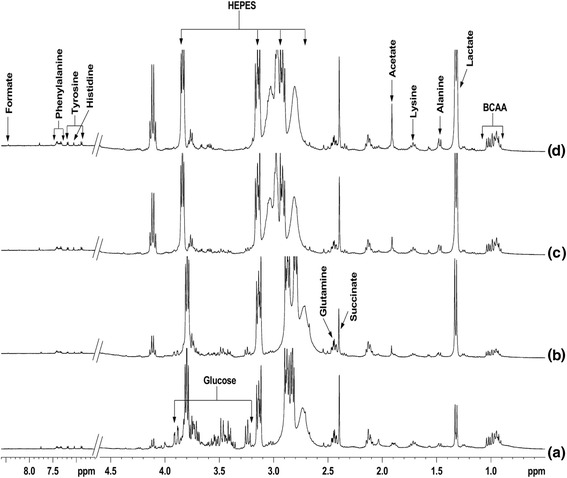
400 MHz 1H NMR spectra of extracellular media of (**a**) 12 h mock infected cells, (**b**) 12 h virus infected cells, (**c**) 24 h virus infected cells, and (**d**) 48 h virus infected cells with E30. All spectra were recorded under identical conditions and plotted with the same vertical scale for direct comparison

**Fig. 3:**
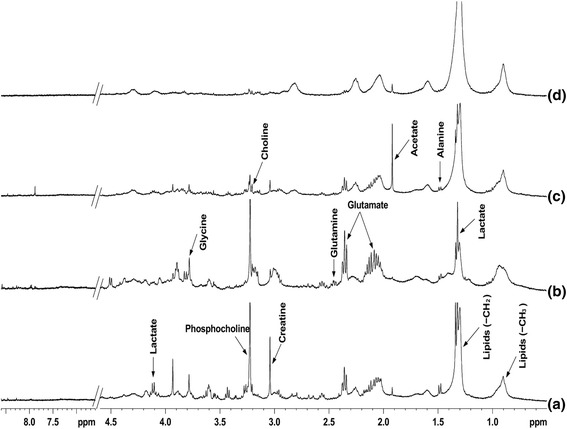
Portions of 400 MHz 1H NMR spectra of RD cells: (**a**) 12 h mock infected cells (**b**) 12 h virus infected (**c**) 24 h virus infected and (**d**) 48 h infected. All spectra were obtained under identical conditions and plotted with identical vertical scale for direct comparison

Spectra of extracellular media from mock infected cells and virus infected cells are shown in Fig. 2. It has possibly well known, in Fig. 2, the quantity of acetate is eminent at 24, and 48 h, post virus infection in comparison with 12 h. In contrast, the quantity of lactate increases gradually from 12 to 48 h post virus infection (Fig. 2). The increased level of acetate at 24 and 48 h may be attributed to the conversion of lactate into acetate. Whereas metabolites like lysine and alanine both were slightly increased from 12 h to 48 h post infection. The amount of succinate was slightly decreased at 12 h infection while no major change was seen at 24 and 48 h post infection. It may be because of normal cell metabolism. Such conversion of lactate to acetate, which accumulated in the growth medium, is reported in several bacterial [19, 20] and viral system [16]. The whole cell spectra showed arised signals of alanine acetate, Creatine, choline, phosphocholine, glycine, and lipids respectively at 12 h mock infected cells (Fig. 3a). The cells after 12 h virus infection showed increased amount of glutamine and glutamate that were not observed in mock infected cells (Fig. 3a). A very high concentration of acetate was observed after 24 h virus infection as compared to mock infected cells as well as 12 h virus infected cells. Whereas the remarkable changes were seen in some metabolites like Creatine, lactate, phosphocholine and glycine were started to decrease gradually at 12 to 48 h post virus infection. At 48-h interval, almost metabolites of infected cells were consumed except some component like lipid and acetate (Fig. 3d).

**Fig. 4:**
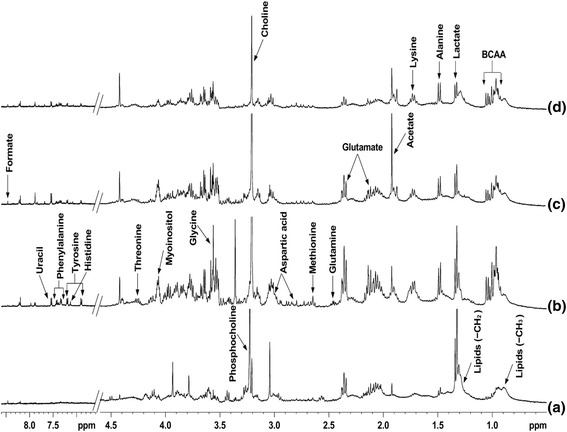
400 MHz 1H NMR spectra of intracellular media of (**a**) mock infected cells, (**b**) 12 h virus infected cells, (**c**) 24 h virus infected cells, and (**d**) 48 h virus infected cells. All spectra were recorded under identical conditions and plotted with the same vertical scale for direct comparison

Figure 4 shows the intracellular metabolites like glutamine, methionine, aspartic acid, glycine, myoinositol, threonine, tyrosine, histidine, uracil, and phenylalanine respectively at 12 h virus infection, however, these metabolites were not seen in mock infected cells. At 24 h infection the glutamine, acetate, and formate were seen, while acetate showed high signals at 24 h and at 48 h. remaining residual metabolites like as lysine, alanine, lactate, and BCAA (Branched chain amino acid) were present at 48 h post infection (Fig. 4d).

**Fig. 5:**
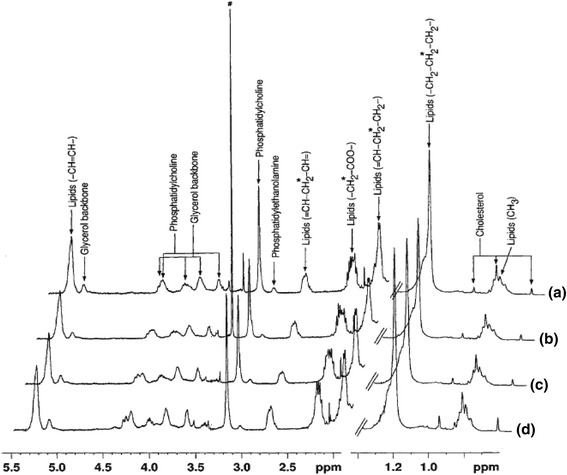
400 MHz 1H NMR spectra of cell lipid components extracted with an organic solvent (chloroform): (**a**) mock infected cells and (**b**)–(**d**) cells infected with E30 for (**b**) 12 h, (**c**) 24 h, and (**d**) 48 h. All spectra were obtained under identical conditions and plotted with the same vertical scale for direct comparison. The peak marked # in 12 h (**b**) refers to methanol impurity

**Fig. 6:**
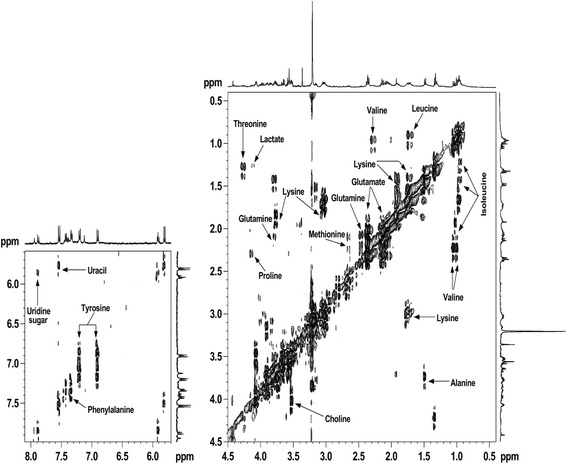
Portion of a 400 MHz COSY spectrum of water-soluble intracellular metabolites of RD cells infected with E30 for 48 h: Alanine, lactate, aspartic acid, valine, leucine, isoluecine, lysine, glutamate, tyrosine, proline, Phenylalanine, Threonine, histidine, and uracil

^1^H NMR spectra of cellular lipids extracted in organic solvent- chloroform, showed signals of lipids, cholesterol, phosphatidylcholine and phosphatidylethanolamine respectively (Fig. 5a-d). The intensities of these lipid signals were increased in virus infected cells as compared to mock infected cells. However, the NMR signals were better determined and more metabolites can be identified, as shown in the 400 MHz DFQ-COSY spectrum of extract from 48 h infected cells (Fig. 6). The recognized metabolites from the two-dimensional (2D) spectra were alanine, valine, leucine, isoleucine, lysine, glutamate, tyrosine, lactate, acetate, aspartic acid, creatine, choline, phosphocholine, phenylalanine, myoinositol, uracil, and formate released respectively. While in case of the spectra of virus infected cells shows (Fig. 3), an increase in viral infection time, all these metabolites gradually reduced at 48 h. In the Figs. 2, 3, 4 and 5 it is clearly seen that amount of cellular lipids increases with increase in E30 infection.

**Fig. 7:**
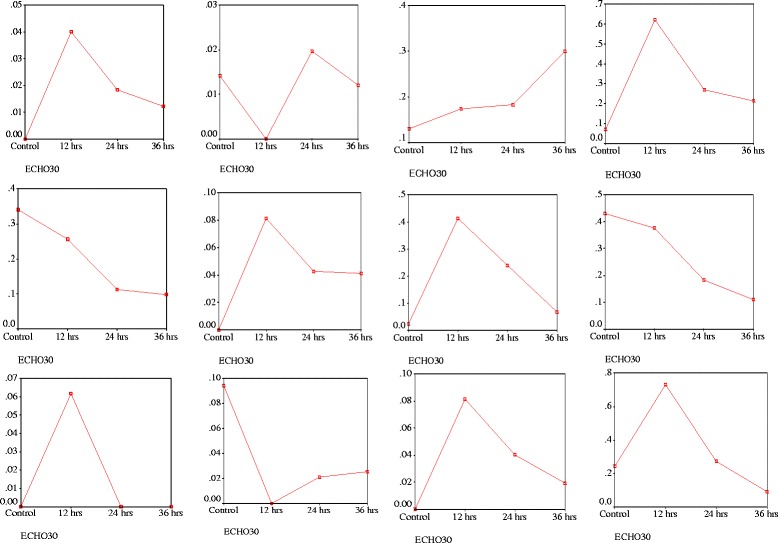
Time interval showing changes in Metabolites after infection in cells

In this study the spectra of lipid extracts for distinctive all time points of mock infected cells and virus infected cells showed major differences in the metabolites. The value of all metabolites gradually decreased during time interval (12, 24 and 48 h) of virus infected cells, whereas mock infected cells utilized the less amount of metabolites corresponding to virus infected cells and released some excretory metabolites during infection as end product (Fig. 7).

## Discussion

A significant association was found between virus infected and mock infected cells at time intervals12, 24, and 48 h for metabolites Alanine, Lactate, Acetate, Glutamate, Tyrosine, Histidine, Phenylalanine, Creatine, Choline and Formate respectively released by cell during infection, which is highly significant (*p* < 0.01). Some insignificant association was also found during the infection period that cells did not use some proportion of metabolites after 12, 24 and 48 h, namely Glutamine and Phosphocholine (Fig. 3).

Cell death through viruses is associated with inhibition of cellular macromolecular synthesis and changes in cellular morphology. The morphological changes are not direct consequences of viral inhibition of host macromolecular synthesis [21]. All cellular components were utilized as time was increased, whereas cell lipid components like phosphatidylcholines, phosphatidylethanolamine, cholesterol, backbone of glycerol and some additional functional group lipid (−CH = CH-), lipid (=CH-CH_2_-CH=), lipid (−CH_2_COO-), lipid (=CH-CH_2_-CH_2_), lipid (−CH_2_-CH_2_-CH), and lipid (−CH_3_) were increased. Thus, these results clearly indicating that EV30 virus does not utilize cell lipids during its viral particle synthesis, in spite of all other cellular components being utilized when cellular damage takes place, as observed by the optical microscopy (Fig. 1). Cell lipid components like, phophatidylethanolamine and phospholipids were also increases in *Adenoviruses* infected on HEK cell along with uninfected cells [22]. Fetal bovine serum in the medium helps the maintenance of viability of the virus to about the same level as observed in medium containing calf serum; therefore, bovine serum was used for this study. The stimulatory effect of sodium oleate on Japanese encephalitis virus growth is clearly shown, where the viral infectivity was almost 50-fold higher in the presence of 9-18:1 (20 μg ml^−1^) than in control medium [23]. It may be noted that E30 does not contain envelop in its outer structure so unable to utilize cell lipids during its viral particle synthesis, 1H NMR results indicate an increase in the cell lipids as time was increased. This hypothesis could account for the increase of enterovirus growth in the presence of fetal bovine serum in the medium, in addition to the virus protective effect of the FBS. There would be one possible reason for increasing concentration of cell lipid with infection time that is the cell death, which is normally an increase in a programmed cell death. A rounding of the cells when come in contact with an antigen and, when mixed in suspension, by the inability of cells to spread normally [24], manifests this phenomenon. Presumably, these effects can be related to changes in cellular membranes and are reflected in the alteration in the lipid metabolism noted.

Interestingly, the intensity of the total lipid signals in the spectra of the cells was similar for the mock infected [25] and 12 h virus infected cells, whereas they gradually increase with virus infection time (Fig. 3). Jenkin and Anderson showed that the sodium salt investigation effect of the fatty acids on viral growth without background interference from the cell [15]. This correlates to the fact that the total quantity of lipid is the same at all time points of mock infected and gradually increased in virus infected cells, as determined from subsequent lipid extraction from whole cells in Fig. 5. It is clearly seen that cell lipid were not utilized by the E30, a non-enveloped virus (Figs. 3, 4 and 5). Unraveling the effects of biological perspective is important only if the intracellular environment can influence the properties of metabolites. The most apparent difference between in-vitro sample and in-vivo is the solute concentration. Macromolecular solutes reach concentrations of hundreds of grams per liter in cells and other biological fluids, but most in-vitro studies are performed in the buffered solution with <1% of the cellular macromolecule concentration. These conditions give optimal signals, but may lack biological relevance [26].

Aspartate and glutamate participate within the neurotransmitter family of substances which play important role in cell. Whereas glutamate is one of the most important excitatory transmitters of central nervous system in lower animals and may also be important in humans. Figure 3 shows that the extracellular metabolites released by cells after infection of E30; At 24 h post virus infection concentration of aspartic acid was high, while it was not observed in mock infected cells and after 48 h virus infection cells were consumed the glutamate and aspartic acid. Glutamate and aspartate are also very important in the TCA cycle. Excitatory transmitters such as aspartate lead to depolarization of the nerves in humans; on the other hand, inhibitory transmitters cause hyperpolarization. When cells showed an apoptotic effect during infection and showed an adverse stress effect. Some of the vital sources like alanine for the production of protein, essential for proper functions of the central nervous system cells and help form neurotransmitters. In-vitro study of uninfected control and infected cell released the alanine in both conditions. Alanine increases during 12 to 24 h post virus infection (Fig. 3) but at 48 h post virus infection it consumed completely. The consumption of alanine is due to readily conversion into glucose when sugar levels fall and amino acids are liberated from cells to provide energy. Alanine may help keep sugar levels stable during virus infection cells. When the energy required for the cells, alanine is converted into the glucose, and thus it may help to maintain the sugar level. Alanine is one of the simplest amino acid and is involved in the energy-production through breakdown of 7glucose. The cells utilized glucose through glycolysis and TCA metabolic pathways to produce several of the metabolic end products such as lactate, acetate and formate respectively into the extracellular medium [16].

*In –vitro* cells study, the creatine was present in the mock infected cells as well as virus infected cells. It was gradually decreased because of the large number of cell death after 48 h infection. The cells get creatine with supplements from minimum essential media (Eagle’s Media) for cell growth. It was interesting that a significant increase in the acetate incorporation into lipids could be obtained even after infection with the virus. The specific activity of the extracted lipid from virus infected cells and mock infected cells are compared. These results suggested that the increased lipid metabolism might have not been induced by the infective virus, but by a component of the virus capsid. It is not known whether this lipid stimulation reflects the formation of membranes necessary for these early events or an introduction to the later synthesis and assembly of particles. It is of interest that a similar increase in lipid synthesis has been described in cells infected with vaccinia [27] and poliovirus [28, 29].

## Conclusion

The present study shows the metabolomics of E30 infected mammalian cells. The NMR analysis revealed no ambiguous signals using the RD cell line. The present proton NMR spectroscopic study, monitoring cellular metabolic changes during virus infection in cells, is useful in probing the molecular mechanism of virus-cell interactions. The fact that the effects of viral infection could be observed through metabolic changes at much earlier stages than previously reported indicates an application for NMR spectroscopy in studying cell-virus interactions. Regardless of the actual mechanism leading to cell shrinkage, it could have significance in late viral functions.

In addition to the consumption of metabolites, the virus-specific RNA and protein synthesis may also be affected by cell shrinkage, the cellular concentration of amino acids, nucleotide concentrations, and other requirements for macromolecular synthesis.

## Abbreviations

CO_2_: Carbon Dioxide
D_2_O: Deuterium Oxide
E30: Echovirus 30
EVs: Enteroviruses
FBS: Fetal Bovine Serum
HCl: Hydrochloric acid
HEPES: N-2-hydroxyethylpiperazine-N0-2-ethanesulfonic acid
KCl: Potassium Chloride
MEM: Minimum Essential Medium
MgCl_2_: Magnesium Chloride
NMR: Nuclear Magnetic Resonance
PBS: Phosphate Buffered Saline
PFU: Plaque Forming Unit
RD: Rhabdomyosarcoma
TSP: TrimethylSilylPropionic acid

## References

[1] Oberste MS, Maher K, Kennett ML, Campbell JJ, Carpenter MS, Schnurr D, Pallansch MA (1999). Molecular epidemiology and genetic diversity of echovirus type 30 (E30): genotypes correlate with temporal dynamics of E30 isolation. J ClinMicrobiol.

[2] Trallero G, Casas I, Tenorio A, Echevarria JE, Castellanos A, Lozano A, Brena PP (2000). Enteroviruses in Spain: virological and epidemiological studies over 10 years (1988-97). Epidemiol Infect.

[3] Lindon JC, Holmes E, Nicholson JK (2004). Metabonomics and its role in drug development and disease diagnosis. Expert Rev Mol Diagn.

[4] Nicholson JK, Wilson ID (2003). Opinion: understanding ‘global’ systems biology: metabonomics and the continuum of metabolism. Nat Rev Drug Discov.

[5] Nicholson JK, Lindon JC, Holmes E (1999). Metabonomics understanding the metabolic responses of living systems to pathophysiological stimuli via multivariate statistical analysis of biological NMR spectroscopic data. Xenobiotica.

[6] Wang Y, Utzinger J, Xiao SH, Xue J, Nicholson JK, Tanner M. System level metabolic effects of a Schistosoma japonicum infection in the Syrian hamster. Mol Biochem Parasitol. 2006;146(1):1–9. 10.1016/j.molbiopara.2005.10.010 16337285

[7] Dagan R, Jenista JA, Menegus MA (1988). Association of clinical presentation, laboratory findings, and virus serotypes with the presence of meningitis in hospitalized infants with enterovirus infection. J Pediatr.

[8] Carrasco L (1994). Entry of animal viruses and macromolecules into cells. FEBS Lett.

[9] Caudai C, Bianchi Bandinelli ML, Lepri A, Valensin PE (1994). Nuclear magnetic resonance investigation of virus-lymphomonocyte interactions. New Microbiol.

[10] Knipe DM, Samuel CE, Palese P, Knipe DM, Howley PM, Griffin DE, Lamb RA, Martin MA, Roizman B, Straus SE (2001). Virus–host cell interaction. Fields Virology.

[11] Mountford CE, Grossman G, Hampson AW, Holmes KT (1982). Influenza virus: an NMR study of mechanisms involved in infection. BiochimBiophysActa.

[12] Stuart AD, McKee TA, Williams PA, Harley C, Shen S, Stuart DI, Brown TD, Lea SM (2002). Determination of the structure of a decay accelerating factor-binding clinical isolate of echovirus 11 allows mapping of mutants with altered receptor requirements for infection. J Virol.

[13] Valensin PE, Bianchi Bandinelli ML, Zazzi M, Gaggelli E, Valensin G (1990). NMR investigation of cell cultures: early detection of infection by herpes simplex virus type 2 and transformation. Microbiologica.

[14] Bissell MJ, White RC, Hatie C, Bassham JA (1973). Dynamics of metabolism of normal and virus-transformed chick cells in culture. Proc Natl AcadSci USA.

[15] Jenkin HM, Anderson LE (1970). The effect of oleic acid on the growth of monkey kidney cells (LLC-MK2). Exp Cell Res.

[16] Tiwari S, Singh RK, Bharti S, Roy R, Singh RK, Dhole TN. An in vitro study- Indian strain of Japanese encephalitis virus infection in porcine stable kidney cell using 1H NMR spectroscopy. International Journal of Experimental Pharmacology. 2012;2(2):50–8.

[17] Kilpatrick DR, Quay J, Pallansch MA, Oberste MS (2001). Type-specific detection of echovirus 30 isolates using degenerate reverse transcriptase PCR primers. J ClinMicrobiol.

[18] Folch J, Lees M, Sloane Stanley GH (1957). A simple method for the isolation and purification of total lipides from animal tissues. J BiolChem.

[19] Talabardon M, Schwitzguébel JP, Péringer P (2000). Anaerobic thermophilic fermentation for acetic acid production from milk permeate. J Biotechnol.

[20] Pintado J, Raimbault M, Guyot JP (2005). Influence of polysaccharides on oxygen dependent lactate utilization by an amylolytic lactobacillus plantarum strain. Int J Food Microbiol.

[21] Eagle H (1959). Amino acid metabolism in mammalian cell cultures. Science.

[22] McIntosh K, Payne S, Russell WC (1971). Studies on lipid metabolism in cells infected with adenovirus. J Gen Virol.

[23] Makino S, Jenkin HM (1975). Effect of fatty acids on growth of Japanese encephalitis virus cultivated in BHK-21 cells and phospholipid metabolism of the infected cells. J Virol.

[24] Russell WC, Hayashi K, Sanderson PJ, Pereira HG (1967). Adenovirus antigens--a study of their properties and sequential development in infection. J Gen Virol.

[25] Rothblat GH, Paoletti P, Kritchevsky D (1969). Lipid metabolism in tissue culture cells. Advances in lipid research.

[26] Charlton LM, Pielak GJ (2006). Peeking into living eukaryotic cells with high-resolution NMR. Proc Natl AcadSci USA.

[27] Gaush CR, Youngner JS (1963). Lipids of virus infected cells. II. Lipid analysis of HeLa cells infected with vaccinia virus. Proc SocExpBiol Med.

[28] Cornatzer WE, Sandstrom W, Fischer RG (1961). The effect of poliomyelitis virus type I (Mahoney strain) on the phospholipid metabolism of the HeLa cell. BiochimBiophysActa.

[29] Penman S (1965). Stimulation of the incorporation of Choline in poliovirus-infected cells. Virology.

