# Erratum to: Metabolomics of rhabdomyosarcoma cell during echovirus 30 infection

**DOI:** 10.1186/s12985-017-0834-1

**Published:** 2017-09-19

**Authors:** Sarika Tiwari, Tapan N. Dhole

**Affiliations:** 1 Department of Microbiology (Virology Section), Sanjay Gandhi Post Graduate Institute of Medical Sciences (SGPGIMS), Uttar Pradesh, Lucknow, -226014 India; 2 Centre for Animal Disease Research and Diagnosis, Indian Veterinary Research Institute, Bareilly, UP India

## Erratum

After publication of the article [1], it has been brought to our attention that the y-axis labels are missing from the graphs shown in Fig. 7. The missing labels are shown in the corrected graphs below. The original version of the article has been revised to reflect this.

**Fig. 7:**
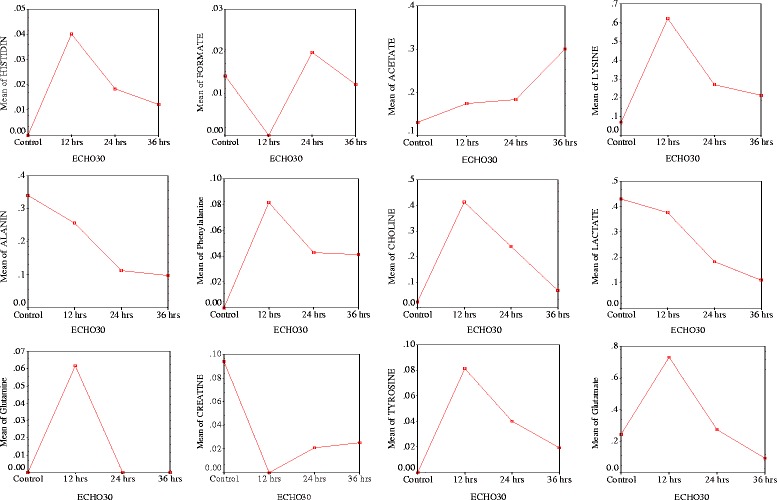
Time interval showing changes in metabolites after infection in cells
